# Analysis of Models to Estimate Morbidity Rates of Respiratory Diseases Through Deep Learning

**DOI:** 10.1111/tmi.70126

**Published:** 2026-03-19

**Authors:** Liliane Moreira Nery, Nícholas de Paula Nicomedes, Pedro Cesar Madureira de Godoy Camargo, Sidney Alves de Outeiro, Leopoldo André Dutra Lusquino Filho, Claudio Miceli de Farias, Darllan Collins da Cunha e Silva

**Affiliations:** ^1^ São Paulo State University (UNESP), Institute of Science and Technology Sorocaba São Paulo State Brazil; ^2^ University of Campinas (Unicamp), Institute of Computing, Recod.AI Lab Campinas São Paulo State Brazil; ^3^ Federal University of Rio de Janeiro (UFRJ) Rio de Janeiro Rio de Janeiro State Brazil

**Keywords:** epidemiological modelling, public health, recurrent neural networks, socioeconomic determinants, time series forecasting

## Abstract

Respiratory diseases remain a challenge in Brazil due to socioeconomic inequalities and environmental risks that intensify population vulnerability. This study compared XGBoost with a deep learning model using stacked Gated Recurrent Units (GRU), trained with morbidity data from respiratory diseases and exogenous variables such as per capita GDP, population density, urbanisation index and greenhouse gas emissions (1999–2023). These data were normalised and temporally disaggregated using synthetic data to refine time‐series granularity. Results showed regional heterogeneity: the GRU achieved superior performance in Porto Alegre (*R* ^2^ = 0.529), São Paulo (*R* ^2^ = 0.518) and São Luís (*R* ^2^ = 0.313), while XGBoost showed mostly negative *R* ^2^ values. These findings demonstrate the potential of recurrent neural networks to capture temporal dependencies in health data and support morbidity forecasting. By anticipating fluctuations, such models can guide resource allocation and inform evidence‐based policies. The study underscores the value of integrating socioeconomic and environmental indicators into predictive frameworks and positions deep learning as a promising tool for precision public health in unequal contexts.

## 1 Introduction

The distribution of respiratory diseases in Brazil is markedly heterogeneous [1], with the effects of these diseases on public health being intensified by socioeconomic and regional inequalities that shape population vulnerability. This occurs due to the unequal distribution of healthcare facilities [2]. The Southern region, for example, has more healthcare facilities per capita than the Amazon region, meaning that Brazil faces difficulties in promoting equity in healthcare services. This inequality in access to healthcare can be seen as an important social determinant [2].

Factors such as the Municipal Human Development Index (MHDI) and the Social Vulnerability Index (SVI) are strongly correlated with mortality rates, showing that the most vulnerable populations are the most affected [3]. For example, the COVID‐19 pandemic, an acute respiratory disease, reinforced this perception, with studies showing that demographic characteristics were determinants for the risk of death [4].

The environmental crisis also aggravates this complex public health scenario [5]. Factors such as accelerated urbanisation, exposure to air pollution and social vulnerability have already been identified as determinants in the spatial distribution of respiratory diseases [6].

It is highlighted [7], for example, that wildfires are a substantial source of pollutants, affecting air quality and human health. Analysing more than 2 million hospital admission records for respiratory and circulatory diseases in Brazil between 2008 and 2018, the authors concluded that epidemiological evidence indicates that air pollution from wildfires and a higher risk of hospital admissions for cardiorespiratory diseases.

Despite evidence on the interaction among socioeconomic factors, pollution and respiratory health [8, 9], the respiratory system constitutes a direct interface for atmospheric pollutants and particulate matter. Consequently, respiratory morbidity exhibits a highly elastic and acute response to environmental stressors, characterised by rapid fluctuations and distinct outbreaks. Brazilian scientific literature still lacks comprehensive analyses that investigate disease prediction in the context of exogenous factors, such as environmental and socioeconomic data.

Given the complexity of these health determinants and the growing volume of available data, traditional statistical methods face limitations, such as missing data, underreporting, redundant data, high dimensionality, heterogeneity and irregularity, which hinder the capture of nonlinear and dynamic patterns. This opens the way for the use of computational deep learning algorithms [10, 11].

While traditional machine learning approaches—such as Support Vector Machines (SVM), Random Forests and autoregressive models (e.g., ARIMA)—have established a strong foundation in predictive modelling, the field has significantly expanded with the emergence of Deep Learning. This advancement, rather than a mere transition, represents a broadening of analytical capabilities driven by the necessity to analyse large volumes of data (big data) and capture complex, non‐linear patterns [11].

Within this landscape, various architectures have been tailored for specific tasks: Convolutional Neural Networks (CNNs) are prominent in image analysis [11], while Recurrent Neural Networks (RNNs), such as Long Short‐Term Memory (LSTM) and Gated Recurrent Units (GRU), are specifically designed to handle sequential data and temporal dependencies [12, 13].

Various deep learning applications are being used to diagnose diseases by interpreting x‐ray images [14], clinical analyses, biomarkers and risk factors [15], Internet of Things and echocardiograms [16] and respiratory diseases symptom [17, 18], as well as to determine risk groups and social determinants that favour increased disease occurrence [4].

Considering the above, the main objective of this study is to develop and evaluate machine learning models for predicting morbidity rates for respiratory diseases in selected Brazilian capitals. The models use morbidity rates along with four exogenous variables: per capita GDP (Gross Domestic Product), population density, urbanisation index and greenhouse gas (GHG) emissions—specifically Methane (CH_4_), Carbon Dioxide (CO_2_) and Nitrous Oxide (N_2_O).

These variables were selected to represent the macro‐structural determinants of health: GDP and urbanisation reflect socioeconomic development and housing conditions, while population density addresses pathogen transmission dynamics. GHG emissions were chosen as a proxy for anthropogenic environmental pressure and industrial activity, linking respiratory health outcomes directly to the primary drivers of climate change, rather than solely to immediate local air quality metrics (such as particulate matter). Justified the choices by the scarcity of studies seeking to predict diseases from exogenous variables, although not directly related to morbidity and mortality rates, exert indirect effects that generate risks [19, 20, 21].

Specifically, the study compares a Gradient Boosting model (XGBoost) with a deep learning model based on a Gated Recurrent Units (GRU) architecture in a stacked configuration. It also aims to: (i) analyse the models' ability to capture trends, peaks and troughs in time series; (ii) discuss the performance differences between the models.

The GRU was chosen for its ability to capture complex temporal dependencies in sequential data, effectively handling nonlinear relationships and retaining relevant information over time. This is possible thanks to its gate structure, which includes an update gate and a reset gate, allowing the GRU to control the flow of information and maintain residual relevant information from the historical data distribution. XGBoost, on the other hand, was selected for its ability to automatically generate important time‐series features, such as lags, time variables (month, year) and moving statistics.

The application of these technologies in building predictive models is fundamentally important for epidemiological surveillance and health management. Accurate predictive models allow for anticipating outbreaks, identifying high‐risk areas and populations and optimising the allocation of healthcare resources.

Filling this gap is essential for developing more effective and targeted public policies. Such an effort aligns with the United Nations Sustainable Development Goals (SDGs), particularly SDG 3 (Good Health and Well‐being) (UN) [22].

This study contributes to the state of the art by contrasting its methodology with existing deep learning approaches in epidemiological modelling. A significant body of literature on infectious disease forecasting primarily utilises machine learning within a univariate context, basing predictions solely on autoregressive patterns derived from the disease's historical time series [23, 24, 25].

When multivariate approaches are adopted, they typically incorporate direct, short‐term causal drivers, with meteorological data being the most common exogenous variables [26]. Although machine learning has been applied to socioeconomic determinants for risk classification—such as in studies on COVID‐19 mortality [20, 27]—the use of machine learning to forecast morbidity time series based on these structural factors remains underexplored.

## 2 Material & Methods

This study adopted a structured methodological framework comprising database construction, spatial delimitation criteria, data preprocessing and the development of predictive models.

### 2.1 Database Construction

A database was created using information provided by the *DATASUS TABNET* system (https://datasus.saude.gov.br/informacoes‐de‐saude‐tabnet/). Monthly morbidity data were collected from 1999 to 2023 for the entire Brazilian territory, concerning Diseases of the Respiratory System (ICD‐10: J00–J99).

Since the identification of groups in some regions is complex due to instability caused by small populations, which are more prone to coefficient fluctuations resulting from rare events, the morbidity rate was calculated as the number of cases per 100,000 inhabitants (Equation 1) [1].

(1) $$\text{Rate}=\left(\frac{n}{\mathrm{pop}}\right)\times 100.000$$

where *n*: number of deaths or number of hospitalised individuals; pop: municipality population.

Data on population, Gross Domestic Product (GDP) and municipal boundaries for the period from 1999 to 2023 were obtained from the Brazilian Institute of Geography and Statistics (IBGE) (https://www.ibge.gov.br/). For population data, IBGE population estimates were used, except for census years (2000, 2010 and 2022) and the population count from the 2007 agricultural census, for which census data were used directly.

The estimated value for the emancipated municipality was subsequently subtracted from the originating municipality's population, ensuring consistency in population totals. The estimated population for time *t*, *P*(*t*), is the sum of the populations of n smaller subregions, *Pi*(*t*), as shown in Equation (2). The determination of the proportionality coefficients (*ai*) and the linear correction term (*bi*) (Equation 3) for estimating *Pi*(*t*) is carried out using demographic census data (Equations 4 and 5).

(2) $$P\left(t\right)=i=1\sum \mathrm{nPi}\left(t\right)$$

(3) $$\mathrm{Pi}\left(t\right)=\mathrm{ai}\cdot t+\mathrm{bi}$$

(4) $$\mathrm{ai}=\frac{\mathrm{Pi}\left(t1\right)-\mathrm{Pi}\left(t0\right)}{t1-t0}$$

(5) $$\mathrm{bi}=\mathrm{Pi}\left(t0\right)-\mathrm{ai}\cdot t0$$

where *Pi*: population of municipality *i*; *P*: population of the federative unit; *t*0: year of census 1; *t*1: year of census 2.

The determination of the municipal urbanisation index and rate was based on urban area data from the MapBiomas Brazil project (https://brasil.mapbiomas.org/). For 11 municipalities with null values, the gaps were filled by estimating urbanised areas for the corresponding years using Landsat 7 satellite images (multispectral resolution of 30 m), after validating the municipality's existence during the study period.

For variables associated with climate change, data on anthropogenic greenhouse gas (GHG) emissions were obtained from EDGARv8.0 (2023) (https://www.edgar.jrc.ec.europa.eu/), a global platform that provides annual emission inventories. Estimates of CH_4_, CO_2_ and N_2_O were collected.

These data were spatially delimited to Brazil and subjected to interpolation using the Inverse Distance Weighted (IDW) method. The pixel size of the resulting raster image was set to 0.01 geographic degrees (~1136.9 m), and the units were standardised from tons of gas/year to kg of gas/km^2^/year, enabling for a more detailed spatial analysis.

### 2.2 Criteria and Data Processing for Delimiting the Study Area

The selection of capitals for this study was a subsequent phase of a broader project, titled “Spatial correlation analysis by deep learning of morbidity and mortality rates with indicators associated with climate and socioeconomic changes” (complete data available at: https://app.powerbi.com/view?r=eyJrIjoiYWI3MmVkMTktMDE3ZC00ZWEzLTg4MzEtMzkwZTlhYzhlMjRjIiwidCI6ImZlODc4N2JjLWM5MTQtNDY2NS04NTQ3LTI2OGUxNWNiMGQ5YSJ9). This larger project encompassed the study of morbidity and mortality from respiratory and circulatory diseases across all 5570 Brazilian municipalities.

**FIGURE 1:**
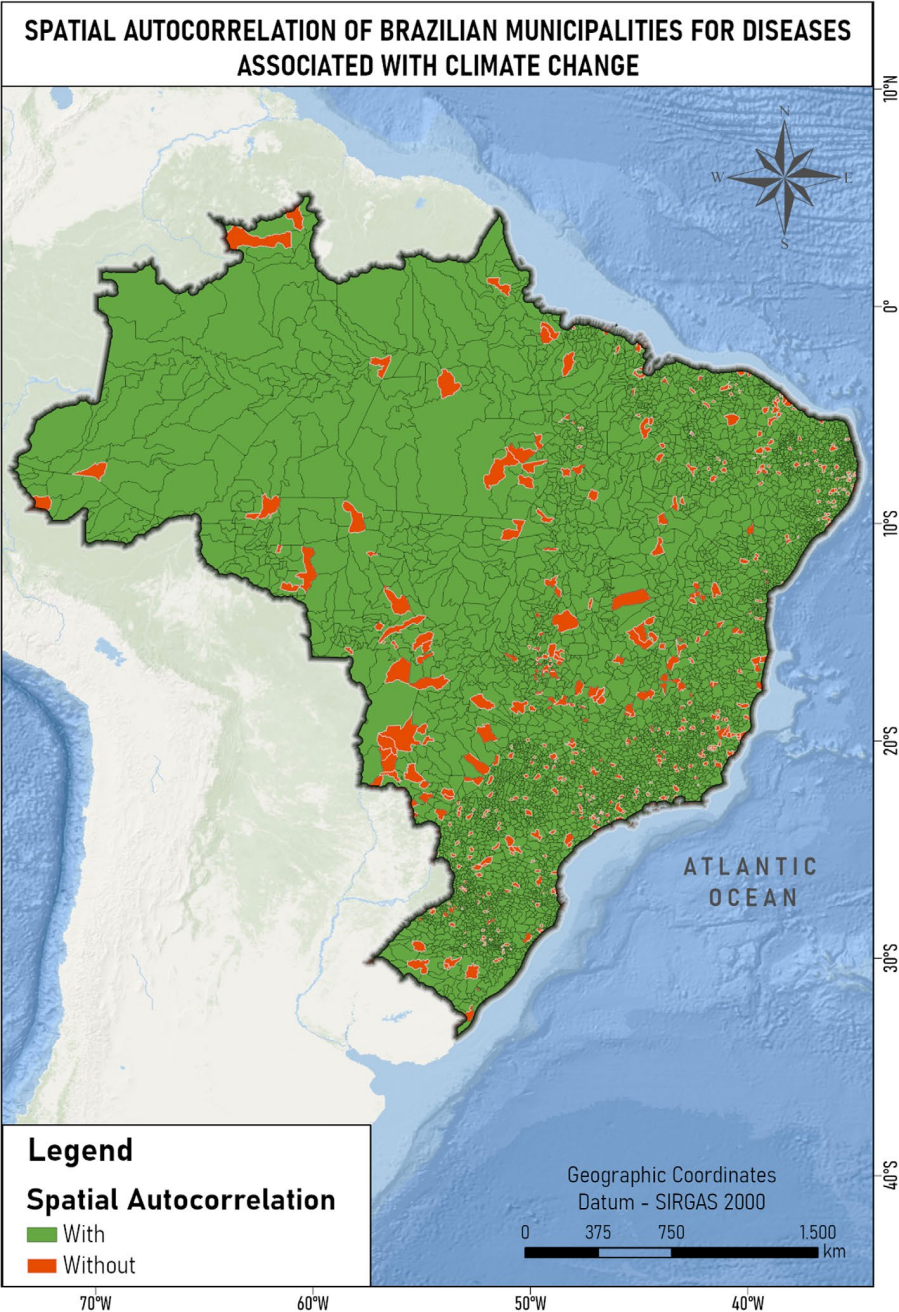
Spatial distribution of Local Moran's I (LISA) clusters for respiratory morbidity in Brazil, where green is municipalities with significant autocorrelation (High‐High/Low‐Low) and red non‐significant areas or spatial outliers excluded from the study.

Within this main project, a Local Moran's I (LISA) analysis was conducted, using 9999 permutations, to identify municipalities with relevant spatial patterns. From this analysis, we identified 5028 municipalities that exhibited statistically significant High‐High (HH) or Low‐Low (LL) spatial autocorrelation for the analysed diseases (Figure 1). The 21 Brazilian capitals included in the present study were part of this subset of 5028 municipalities.

So, only municipalities that showed significant spatial autocorrelation of the High‐High (HH) or Low‐Low (LL) type for morbidity rates from respiratory diseases were included. This approach ensured that projections were made only for locations with spatial patterns relevant to the health variables under study, avoiding the introduction of biases that could arise from including capitals without this spatial characteristic.

### 2.3 Data Organisation and Dependencies

The analysis environment was configured in a Jupyter Notebook, divided into sections that segment each stage of the process. All files were loaded and stored in a Python dictionary, allowing for efficient programmatic access and manipulation. This structure also enables operations such as DataFrame merges, year extraction and data conversions.

### 2.4 Normalisation (z‐Score)

To allow socioeconomic variables to be combined, z‐score normalisation was applied, as defined in Equation (6). This ensured that variables with different scales were comparable.

(6) $$x_{i}^{*}=\frac{\left(x_{i}-\mu_{x}\right)}{\sigma_{x}}$$

where $x_{i}$ is the original value of the variable; $\;\mu_{x}$ is the mean value of the variable; $\;\sigma_{x}$ is the standard deviation of the variable.

### 2.5 Morbidity Projection Models

For the time‐series analysis of the rates studied here, the original monthly frequency of the data proved a limitation for training the tested algorithms. Therefore, it was necessary to perform temporal disaggregation, transforming monthly data into weekly data.

This procedure increases the temporal resolution of the data series, allowing for a more detailed and dynamic analysis. A standard approach for analysing small datasets is Data Augmentation (DA), which generates synthetic data to expand the original dataset while attempting to remain close to its probability density function [28]. For this purpose, Jittering (Equation 7) and Cubic Spline (Equation 8) methods were used.

(7) $$x^{\prime }\left(\epsilon \right)=\left\{x_{1}+\epsilon_{1},\backslash \text{dots},x_{t}+\epsilon_{t},\ldots ,x_{T}+\epsilon_{T}\right\}$$

(8) $$S\left(x\right)=\left\{\begin{array}{c} \begin{array}{cc} S_{1}\left(x\right) & \text{if}\;x_{1}\leq x<x_{2}\\ S_{2}\left(x\right) & \text{if}\;x_{2}\leq x<x_{3}\\ \vdots & \vdots \end{array}\\ S_{n-1}\left(x\right)\;\text{if}\;x_{n-1}\leq x<x_{n} \end{array}\right.$$

where *ϵ* corresponds to the white noise used in Jittering; x is the original point, in this case corresponding to the monthly data (dataset); S is the third‐degree polynomial defined by $s_{i}\left(x\right)=a_{i}{\left(x-x_{i}\right)}^{3}+b_{i}{\left(x-x_{i}\right)}^{2}+c_{i}\left(x-x_{i}\right)+d_{i}$.

Jittering is commonly used in the context of DA, applying Gaussian white noise with a standard deviation of 0.1 for dataset normalisation, given that the temporal resolution was changed from annual to monthly [29, 30, 31].

The cubic spline method, in turn, uses interpolation that fits a distinct third‐degree polynomial for each interval between sequential data points [32].

Since no ground‐truth weekly morbidity series are available for Brazilian municipalities, the validation of the temporal disaggregation procedure was performed using a distributional consistency analysis rather than pointwise comparison. The objective was to assess whether the synthetic temporal refinement introduced distortions in the statistical properties of the original signal.

For each municipality, a two‐sample Kolmogorov–Smirnov (KS) test was applied to compare the empirical distribution of the original monthly morbidity series with that of the synthetically generated series after resampling back to the monthly scale. The null hypothesis (*H*_0_) assumes that both samples originate from the same underlying distribution. A *p* value greater than or equal to 0.05 indicates insufficient evidence to reject *H*_0_, suggesting that the interpolation process preserves the distributional characteristics of the original data.

The results indicate that approximately 90% of the municipalities presented KS test *p* values ≥ 0.05, demonstrating that the combined jittering and cubic spline interpolation did not significantly alter the statistical distribution of morbidity rates in the majority of cases. This supports the validity of using synthetically disaggregated weekly data for model training, as the procedure preserves the empirical characteristics of the original monthly time series while increasing temporal resolution.

To predict health indicators, two types of algorithms were used. First, a stacked gated recurrent unit (GRU) neural network was applied. It is worth noting that a single‐layer neural network (NN) consists of: (1) receiving input data; (2) multiplying them by weights; (3) summing these values; (4) calculating the difference between the weighted sum of inputs and the activation threshold (bias); (5) generating the output data according to an activation function [33, 34].

**FIGURE 2:**
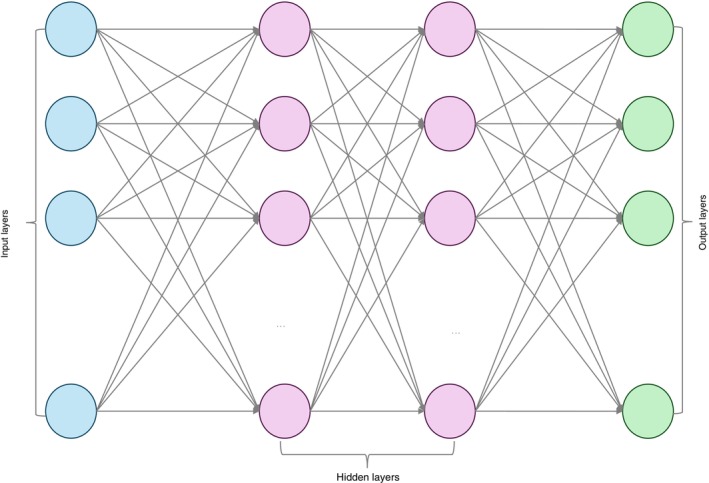
Schematic of the multi‐layer neural network architecture with the data flow from the input layer through stacked hidden layers for feature extraction to the final prediction output.

A multi‐layer NN structure (Figure 2) is divided into: (1) input layers, which in our model receive the tensors containing the historical morbidity rates and exogenous variables (e.g., GDP, GHG emissions); (2) hidden layers, responsible for processing these features to capture non‐linear temporal patterns and (3) output layers, which generate the final morbidity prediction. The connections between these layers are defined by weights (nodes), representing the learned strength of the relationships between the input variables and the health outcome [35].

In this context, the stacked GRU model can capture temporal patterns, such as the evolution of disease cases, while preserving the influence of information from distant periods in the past and reducing overfitting.

The NN received time sequences in the form of tensors—a multilinear map that gathers many linear objects—used in machine learning to generate a set of features from data formatted within an array of one or more dimensions, with dimensions [batch_size, seq_len, n_features], where each sample represents 1 month of the analysed data. Its architecture includes two stacked LSTM layers, each containing 64 units in the hidden layer and dropout (a technique used during training that randomly turns off neurons in each epoch to avoid overfitting) set to 0.2.

The model outputs are structured into two neurons: (1) Classification—A linear layer generates a single value (logit), estimating the probability of an event occurrence (rate greater than zero), trained with the *BCEWithLogitsLoss* loss function; (2) Regression—Another linear layer provides the continuous rate prediction, applied only to cases with event occurrence. This output was trained with *MSELoss*.

To optimise training, early stopping and adaptive learning rate adjustment (*ReduceLROnPlateau*) techniques were incorporated, allowing interruption when validation performance did not improve and dynamic learning rate adjustments to refine model parameters.

The models were evaluated based on the coefficient of determination (*R* ^2^), root mean square (RMS) and root mean square error (RMSE). After applying the stacked GRU model, the best results (highest *R* ^2^, lowest RMS and RMSE) were assessed for later comparison with the XGBoost (*eXtreme Gradient Boosting*) model, a tree‐based approach that includes the generation of lag variables (past values of the series itself), the incorporation of temporal variables (such as month, year or day of the week), and the calculation of moving statistics (such as means or standard deviations over defined time windows).

The fundamental distinction between the two architectures lies in how they handle temporal information. The term ‘recurrent’ in GRU refers to the network's ability to process data as a continuous sequence rather than isolated observations. Through its internal loops, the GRU maintains a ‘hidden state’ (memory) that is updated at each time step, allowing historical information to naturally persist and influence future predictions.

In contrast, XGBoost, being a tree‐based model, treats time series data as a standard tabular problem. It does not possess an internal state to track time; instead, it relies on ‘feature engineering’ (such as manually created lag variables) to interpret past contexts.

Socioeconomic data, including per capita GDP, population density, urbanisation index and GHG emissions (CH_4_, total CO_2_ and N_2_O), were incorporated on an annual scale due to the availability of this information in that format.

To reconcile the different temporal levels, the annual values were transformed into long format and integrated into the weekly dataset. Thus, each municipality came to have a weekly time‐series in which the annual socioeconomic variables were replicated for the weeks of the respective year.

To preserve temporal causality and avoid information leakage, model validation was conducted using strictly chronological train–validation–test splits rather than random sampling. Time series data were partitioned according to their natural temporal order, ensuring that all observations used for training precede those used for validation and testing.

Specifically, for each municipality, the weekly time series was divided into three contiguous subsets: the first 70% of observations were used for model training, the subsequent 20% for validation and hyperparameter tuning and the final 10% for out‐of‐sample testing. This protocol simulates a realistic forecasting scenario, in which future morbidity rates are predicted exclusively from past information.

Model performance was evaluated on the held‐out test set using MAE, RMSE, symmetric Mean Absolute Percentage Error (sMAPE) and the coefficient of determination (*R* ^2^). The use of multiple error metrics allows for a robust assessment of predictive accuracy, sensitivity to extreme values and relative error behaviour across municipalities with distinct morbidity scales.

Although traditional k‐fold cross‐validation is widely used in machine learning, it was not adopted in this study because it violates the temporal dependency structure inherent to epidemiological time series, potentially introducing look‐ahead bias. The adopted chronological splitting strategy ensures temporal integrity while providing a consistent and comparable evaluation framework across all municipalities.

Performance consistency across multiple municipalities with heterogeneous epidemiological profiles further supports the robustness of the adopted validation strategy.

To capture temporal dynamics, windowing was applied, transforming each municipality's time‐series into fixed‐size sequences (e.g., 52 consecutive weeks), allowing the GRU network to learn seasonal patterns and integrate contextual information from the annual variables (GHG, per capita GDP, population density and urbanisation index).

The proposed hybrid approach begins with one or more GRU layers, that extract latent representations from the time‐series. The output of this stage is then fed into two distinct neurons: one for event detection (classification of the morbidity rate as zero or positive) and another for rate prediction (regression) in cases when the event occurs. This structure allows for differentiated handling of periods with absence and presence of cases, a critical aspect given the high proportion of zeros in the dataset.

## 3 Results

### 3.1 Analysis of Predictive Models With Stacked GRU

**FIGURE 3:**
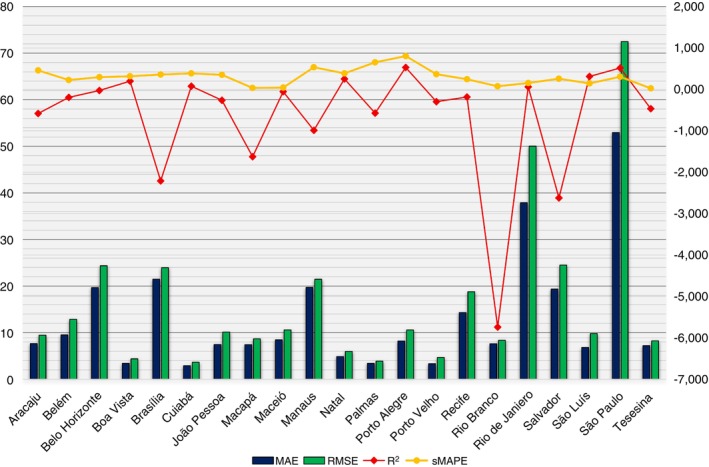
Performance metrics of the Stacked GRU model across 21 Brazilian capitals: Coefficient of Determination (*R* ^2^), Root Mean Square (RMS), Root Mean Square Error (RMSE) and Symmetric Mean Absolute Percentage Error (sMAPE) highlighting regional performance heterogeneity. The horizontal axis represents the analyzed capitals, while the vertical axis displays the metric values.

Figure 3 presents the organised values of *R* ^2^, RMS and RMSE, demonstrating the model's performance for the 21 Brazilian capitals analysed. A wide variability in predictive performance among cities is observed, which can be attributed to Brazil's socioeconomic and environmental heterogeneity.

Capitals such as Porto Alegre (RMS: 8.20; RMSE: 10.57; *R* ^2^: 0.529) and São Luís (RMS: 6.81; RMSE: 9.78; *R* ^2^: 0.313) presented the best positive *R* ^2^ results, indicating that the model was able to explain a considerable proportion of the variability in the observed data.

Despite high RMS (52.96) and RMSE (72.50) values, São Paulo shows a positive *R* ^2^ (0.517), similar to that of Porto Alegre. This suggests that although the model was able to capture the general trend and variability of São Paulo's data (resulting in a high *R* ^2^), the magnitude of absolute prediction errors is considerable due to the higher morbidity rate resulting from the population density of this large metropolis. Consequently, even a small percentage error can translate into a significant absolute error.

Curitiba (RMS: 2.89; RMSE: 3.64; *R* ^2^: 0.078) also showed a positive *R* ^2^, although it is considerably lower when compared to Porto Alegre (*R* ^2^ = 0.529), São Paulo (*R* ^2^ = 0.517), São Luís (*R* ^2^ = 0.313), Natal (*R* ^2^ = 0.255) and Boa Vista (*R* ^2^ = 0.202). These cities may have more consistent temporal patterns or be less susceptible to atypical fluctuations, facilitating prediction.

On the other hand, most capitals studied presented negative *R* ^2^ values. Salvador (*R* ^2^: −2.621), Rio Branco (*R* ^2^: −5.74) and Brasília (*R* ^2^: −2.209) exhibited the worst performances in terms of *R* ^2^, suggesting that the predictive model was unable to capture variability.

This may indicate greater complexity or randomness in the epidemiological patterns of these locations, the presence of extreme events not captured by the model, to include more specific, context‐specific exogenous variables to improve predictive capacity.

Regarding error analysis, cities with lower RMS/RMSE values generally correspond to those with higher positive *R* ^2^ values (notably Porto Alegre and São Luís), while cities with negative *R* ^2^ values tend to have higher RMS and RMSE values, reflecting low accuracy in some regions.

### 3.2 Comparative Analysis Between Stacked GRU and XGBoost

**FIGURE 4:**
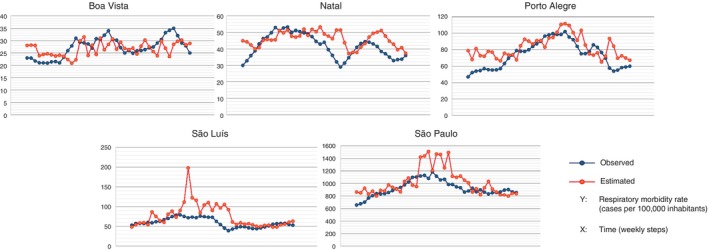
XGBoost time series forecasting for respiratory morbidity to compare observed rates (ground truth) against model predictions for representative capitals, showing limited capture of peak magnitudes. The horizontal axis represents the timeline (weeks), and the vertical axis indicates the standardized morbidity rate. [Correction added on 16 April 2026, after first online publication: The legend for Figure 4 has been corrected to read “Y: Respiratory morbidity rate (cases per 100,000 inhabitants).”]

**FIGURE 5:**
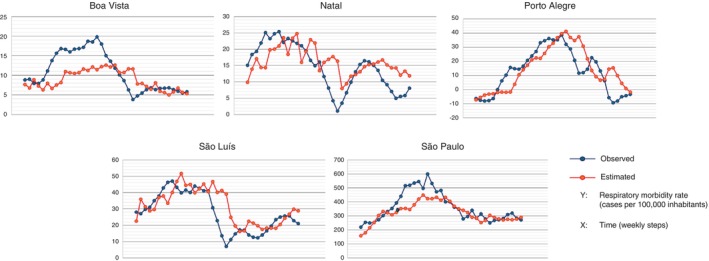
Stacked GRU time series forecasting for respiratory morbidity to compare observed rates (ground truth) against model predictions, demonstrating the model's ability to capture temporal dependencies and seasonal peaks. The horizontal axis represents the timeline (weeks), and the vertical axis indicates the standardized morbidity rate. [Correction added on 16 April 2026, after first online publication: The legend for Figure 5 has been corrected to read “Y: Respiratory morbidity rate (cases per 100,000 inhabitants).”]

When comparing the forecasting models, notable differences in the and response can be observed between the stacked GRU and XGBoost. XGBoost shows more pronounced oscillations during estimates (Figure 4), while the stacked GRU presents smoother oscillations (Figure 5).

The lower predictive capacity of XGBoost is evident in Table 1, where only Porto Alegre shows a positive *R* ^2^. The models demonstrate a general ability to capture seasonality and long‐term trends in the time series. However, the GRU model has a greater ability to react more quickly to fluctuations and peaks, particularly in Porto Alegre, São Luís and São Paulo.

**TABLE 1:** Comparative predictive performance (MAE, RMSE, sMAPE and *R* ^2^) between Stacked GRU and XGBoost models for Brazilian capitals; the bold values indicate positive *R* ^2^, signifying performance superior to a baseline average prediction.

Municipality	XGBoost				Stacked GRU			
	MAE	RMSE	*R* ^2^	sMAPE	MAE	RMSE	*R* ^2^	sMAPE
Porto Alegre	12.387	15.122	**0.142**	16.93%	8.204	10.572	**0.529**	80.45%
São Paulo	137.159	182.868	−1.193	13.28%	52.963	72.503	**0.518**	14.64%
São Luís	18.727	30.005	−6.075	23.71%	6.814	9.787	**0.313**	25.91%
Natal	6.118	7.997	−0.279	1.46%	4.87	5.971	**0.255**	38.76%
Boa Vista	3.367	4.176	−0.101	12.61%	3.405	4.382	**0.202**	31.73%

The comparative analysis between the models, detailed in Table 1, demonstrates the superiority of the stacked GRU architecture over XGBoost, as the stacked GRU model performed better across practically all the capitals evaluated.

For cities such as Porto Alegre, São Paulo and São Luís, the GRU achieved considerably lower errors (MAE and RMSE) and, crucially, a positive coefficient of determination (*R* ^2^), in contrast to the negative values obtained by XGBoost. This confirms the recurrent neural network model's greater ability to adapt to the complexity of health time series, justifying its selection for subsequent analyses.

## 4 Discussion

The high performance of the stacked GRU model in cities such as Porto Alegre (*R* ^2^ = 0.529) and São Paulo (*R* ^2^ = 0.518), and the negative *R* ^2^ values in capitals of the North and Northeast, such as Salvador (*R* ^2^ = −2.621), Belém (−0.192) and Rio Branco (−5.741), as observed in Figure 2, suggest a geographical pattern. Even with a uniform set of exogenous variables (GDP, urbanisation, population density and GHG emissions), a marked heterogeneity in model performance was observed.

When analysing circulatory diseases, a downward mortality trend was identified in the South and Southeast regions, while the Northeast showed an upward trend, and a strong correlation was found between the Municipal Human Development Index (MHDI) and mortality (Pearson's coefficient of −0.89) [3].

Thus, our set of exogenous variables (GDP, urbanisation, population density and GHG emissions) was insufficient to capture the complex network of social determinants governing health in these regions, which influence epidemiological patterns [3]. The absence of a social vulnerability indicator in our model is a probable explanation for its low generalisation capacity in the North and Northeast regions, leading to high errors and, consequently, negative *R* ^2^ values.

To statistically investigate this heterogeneity, a correlation analysis was conducted, cross‐referencing the model's performance (GRU *R* ^2^ values) with the mean profile of the exogenous variables used in training (1999–2023) for each capital. This approach maintains methodological coherence by assessing whether the model's performance correlates with the same variables it utilised.

The GRU's performance (*R* ^2^) exhibited a moderate positive correlation with the Urbanisation Index (*r* = 0.3986) and Population Density (*r* = 0.3331) (observed in Table 2), contrasting sharply with the weak correlation found with GDP per capita (*r* = 0.0773). This suggests that economic distribution had little influence on the model; rather, the structural context of urbanisation and territorial occupation was more determinant. This statistical evidence demonstrates that the GRU model generalised better in denser, more urbanised capitals (predominantly in the South and Southeast regions) and failed in locations with other profiles.

**TABLE 2:** Pearson correlation (r) between the GRU model's performance (*R* ^2^) and the mean values of exogenous socioeconomic/environmental variables (1999–2023) across 21 capitals.

Variables	*r*
Demographic density	0.3331
Urbanisation index	0.3986
Gross domestic product	0.0773
Methane	0.1997
Carbon dioxide	0.2535
Nitrous oxide	0.2633

For Porto Alegre, the XGBoost model already showed promising results compared to the other municipalities (Figure 3). Yet, the series estimated by the stacked GRU followed the real data more faithfully, which is corroborated by a high coefficient of determination (*R* ^2^ > 0.529). The GRU also showed greater ability to reach incidence peaks, suggesting a robust and consistent fit.

In São Luís and Boa Vista, a significant improvement in predictions was observed with the use of the GRU (Figure 4), as for the former, the XGBoost model constantly overestimated the rates (resulting in the worst *R* ^2^, as seen in Table 1). In contrast, the opposite was observed for Boa Vista (Figure 3).

The predictions generated by the GRU aligned more closely with the real data, showing a more accurate response to abrupt variations and suggesting superior fit in scenarios with greater variability, as also seen for Natal (Figure 4). The analysis for São Paulo revealed that the XGBoost model produced considerable errors (MAE/RMSE), as verified in Table 1.

Although MAE and RMSE values were also high for the GRU model, it provided a more robust response, replicating to some extent the peaks and troughs of the rates—in other words, the model produced predictions close to the ground truth in terms of time‐series similarity. Despite moments of underestimation and overestimation, the ability to follow the series' general trend was maintained and potentially improved.

The superiority of the stacked GRU over XGBoost, as observed across all metrics for cities such as São Paulo and Porto Alegre, highlights the importance of model architecture in health time‐series analysis. Although XGBoost is described in the literature as a resilient and highly accurate model for health prediction [13, 14], its performance in our study was limited, with positive *R* ^2^ only for Porto Alegre.

This suggests that, although effective for classification problems, its decision‐tree–based structure may be less suited to capturing long‐term temporal dependencies and complex seasonal patterns present in morbidity data.

The superior ability of the stacked GRU model to respond to fluctuations and incidence peaks, especially in Porto Alegre, São Luís and São Paulo, is also directly attributable to its structural characteristics. Architectures such as GRU and LSTM (long short‐term memory) are specifically designed to process sequential data, with learning capacities that allow them to capture complex time series [11].

It has been concluded that LSTM models are more accurate in predicting peak disease occurrences and perform better when the number of cases is rapidly increasing^37^. Furthermore, it can be inferred that stacking enhanced this capacity by combining the GRU's temporal pattern‐capturing ability with the generalisation capacity of other models, refining predictions and improving the final fit.

The stacked GRU model demonstrated a statistically superior fit in high‐variability scenarios. In capitals such as Porto Alegre and São Paulo, the GRU achieved *R* ^2^ values of 0.529 and 0.517, respectively, indicating it successfully explained over 50% of the data variance. In sharp contrast, the XGBoost model failed to capture this variability, resulting in negative *R* ^2^ values across nearly all capitals, except Porto Alegre (Table 1). This quantitative evidence confirms that the GRU provided a more robust response in replicating the series, including its peaks and troughs, making it more suitable for understanding and replicating disease spread dynamics [28].

Despite the results, this study has limitations that open clear avenues for future research. First, the set of exogenous variables, although relevant, was limited. For example, a previous study demonstrated the substantial impact of particulate matter (PM2.5) from wildfires on respiratory hospitalizations, a more specific pollutant than GHG emissions data, and one that could enhance the model [7]. Other data, such as the SVI, could also help balance predictive accuracy, turning these models into robust tools for epidemiological surveillance and precision public health planning in Brazil and in other countries with marked inequalities.

Another point is the temporal granularity of our data. Although we disaggregated monthly data into weekly data through data augmentation, previous studies, such as those that used daily data, allow for a finer capture of abrupt fluctuations and peaks [28].

However, the incorporation of environmental and socioeconomic variables is a distinctive feature of this study, which, by adopting a multivariate approach, advances the attempt to model the complex network of determinants influencing respiratory health in a country with multiple contexts. Therefore, despite the limitations presented, the relevance of our approach with exogenous variables—which help contextualise trends on a broader temporal scale—should not be diminished, as it provides promising directions for future investigations that could combine other data sources.

Finally, it is worth noting the possibility of continuing this research by comparing these models with other auto‐regressive methods that support exogenous variables, such as SARIMAX, and with contemporary predictors such as transformers and N‐HiTS.

### 4.1 Applications for Public Health

The results obtained have direct implications for public health management, as they contribute to the validation of a tool that can serve as a basis for preventive responses to epidemiological crises grounded in real‐world data. This aligns with the broader objectives of epidemiological modelling, which seeks to provide actionable foresight for infectious disease control, as observed in Chae et al. (2018) and Sebastianelli et al. (2024).

The successful implementation of these models serves as a proof of concept for the discussion of deploying epidemiological early warning systems. In practice, a model achieving *R* ^2^ values such as those observed in Porto Alegre (*R* ^2^ = 0.529) and São Paulo (*R* ^2^ = 0.517) can generate reliable forecasts weeks in advance. Consequently, health secretariats could use these predictions to trigger graduated alert levels, facilitating the organisation and initiation of campaigns targeted at high‐risk populations, intensifying precautions and placing primary care units on alert.

The GRU's more accurate peak forecasting provides a quantitative basis for acute hospital planning, enabling managers to proactively manage bed capacity, schedule medical staff, postpone elective surgeries and ensure the integrity of the hospital supply chain, thereby mitigating system collapses during the peak of the respiratory disease season. Furthermore, despite its limitations and challenges, the integration of socioeconomic and environmental variables offers a more holistic forecast than simple autoregressive models, allowing state and federal agencies to anticipate resource allocation on a regional scale.

Finally, the model's failure in the North and Northeast regions (e.g., Salvador: *R* ^2^ = −2.621; Rio Branco: *R* ^2^ = −5.741) provides statistical evidence that a ‘one‐size‐fits‐all’ national predictive model is unviable. This finding strongly corroborates our hypothesis that the initial set of exogenous variables was insufficient to capture the complex health determinants in these areas. This aligns with literature demonstrating deep socioeconomic and spatial disparities in healthcare access in Brazil (Bastos et al. 2022; Carabali et al. 2020). Consequently, the most significant practical implication of these results is the need for regionalized and data‐driven policies. This supports a shift towards ‘precision public health’, localised, context‐specific models guide resource allocation and interventions.

## 5 Conclusion

The deep learning model (stacked GRU) proved significantly superior to the machine learning model (XGBoost) for predicting morbidity rates for respiratory diseases, especially in cities with high variability (peaks and troughs throughout the historical series), such as Porto Alegre, São Paulo and São Luís.

The considerable variation in results suggests that the variables used (GDP, urbanisation, population density and GHG emissions) were not sufficient to capture the socioeconomic and health‐specificities of each city. Local factors not included in the model appear to have a significant influence on the observed heterogeneity. Specifically, structural determinants such as the Social Vulnerability Index (SVI) and disparities in access to healthcare services are likely decisive in less urbanised regions. For major urban centers like São Paulo, the incorporation of direct Air Quality Index (AQI) data, such as those provided by environmental agencies, could refine the model by capturing acute exposure to pollutants more effectively than annual GHG emissions. Furthermore, complementing per capita GDP with inequality indicators, such as the Gini Index, would mitigate economic dispersion biases, allowing the model to identify susceptible population clusters even within wealthier municipalities.

## Funding

This work was supported by Coordenação de Aperfeiçoamento de Pessoal de Nível Superior, 001; Conselho Nacional de Desenvolvimento Científico e Tecnológico, 444734/2023‐6.

## Conflicts of Interest

The authors declare no conflicts of interest.

## Data Availability

The data that support the findings of this study are available from the corresponding author upon reasonable request.
